# Short-term changes in radiographic joint space width after jiggling exercise as conservative treatment for hip osteoarthritis: A retrospective case series of nine patients

**DOI:** 10.1371/journal.pone.0253643

**Published:** 2021-06-22

**Authors:** Hisayoshi Yoshizuka, Takanori Sato, Junya Murakami, Tsubasa Mitsutake, Masao Hiromatsu

**Affiliations:** 1 Department of Physical Therapy, Fukuoka International University of Health and Welfare, Fukuoka City, Fukuoka, Japan; 2 Department of Rehabilitation Medicine, Yanagawa Rehabilitation Hospital, Yanagawa City, Fukuoka, Japan; 3 Department of Orthopedic Surgery, Yatsushiro Keijin Hospital, Yatsushiro City, Kumamoto, Japan; Prince Sattam Bin Abdulaziz University, College of Applied Medical Sciences, SAUDI ARABIA

## Abstract

Jiggling exercise is a conservative treatment for hip osteoarthritis, which involves continuous shaking of the foot and leg in small oscillations while seated. Previous studies have shown beneficial effects of jiggling exercises for outpatients with advanced- and terminal-stage hip osteoarthritis when performed for longer than 1 year, including increases in joint space width and remission of symptoms. We aimed to use the data from our own treatment to evaluate the short-term impact of intensive jiggling exercises on inpatients with hip osteoarthritis to further examine the clinical utility of this exercise. This retrospective case series study included nine patients (57 ± 12 years) with nine hip joints with advanced- or terminal-stage hip osteoarthritis who performed continuous daily jiggling exercises, beginning from day of hospitalization to 6 months post-discharge. Jiggling exercise was performed seated, using the KENKO YUSURI^®^ automated heel vibrating machine at 3.3–5.0 Hz. The patients were also instructed against weight-bearing during hospitalization. The values of radiographic joint space width and Japanese Orthopaedic Association hip score for pain at hospital admission, discharge, and at the 6-month post-discharge checkup were evaluated. Although the hospitalization period and daily time spent performing the jiggling exercise varied in each case (27–98 days and 2–6 hours, respectively), the joint space width increased in all patients and there was an improvement in the hip pain scores in eight patients. The mean values of the minimum joint space width and hip pain scores at discharge were the highest compared to those at hospital admission and 6 months post-discharge. Our results suggest that intensive jiggling exercise for inpatients with advanced- and terminal-stage hip osteoarthritis leads to earlier improvement in joint space width and pain. Daily jiggling exercise for an adequate duration or in combination with non-weight-bearing practices may be a feasible conservative treatment for hip osteoarthritis.

## Introduction

Hip osteoarthritis (OA) is the most common joint disease and a leading cause of chronic pain, contributing to limited range of motion, walking, and activity. In 2017, approximately 2 million new patients were diagnosed with hip OA globally, bringing the number of individuals worldwide suffering from the condition to ~40 million [1]. The recent estimate of global years lived with disability due to hip OA reached 1.26 million in 2017, which was an increase of 35.3% from 2007 [1]. Due to its progressive and chronic nature, hip OA requires remarkable healthcare resources and involves considerable social costs for treatment, and these demands are bound to increase further in an aging population [2].

The widely recommended guidelines for the treatment of hip OA include non-pharmacological methods such as patient education and self-management, exercise therapy, weight loss in case of overweight or obese individuals, and walking aids as indicated, which are commonly applied as first-line treatment [3, 4]. Though joint replacement surgery is a clinically relevant and cost-effective treatment for terminal-stage hip OA, the surgery should be considered only if all appropriate conservative options, delivered for at least 6 months, have been unsuccessful [5]. However, at present, there are no established effective disease-modifying non-invasive interventions that can prevent, halt, or restrict the progression of OA [5–7].

**Fig 1:**
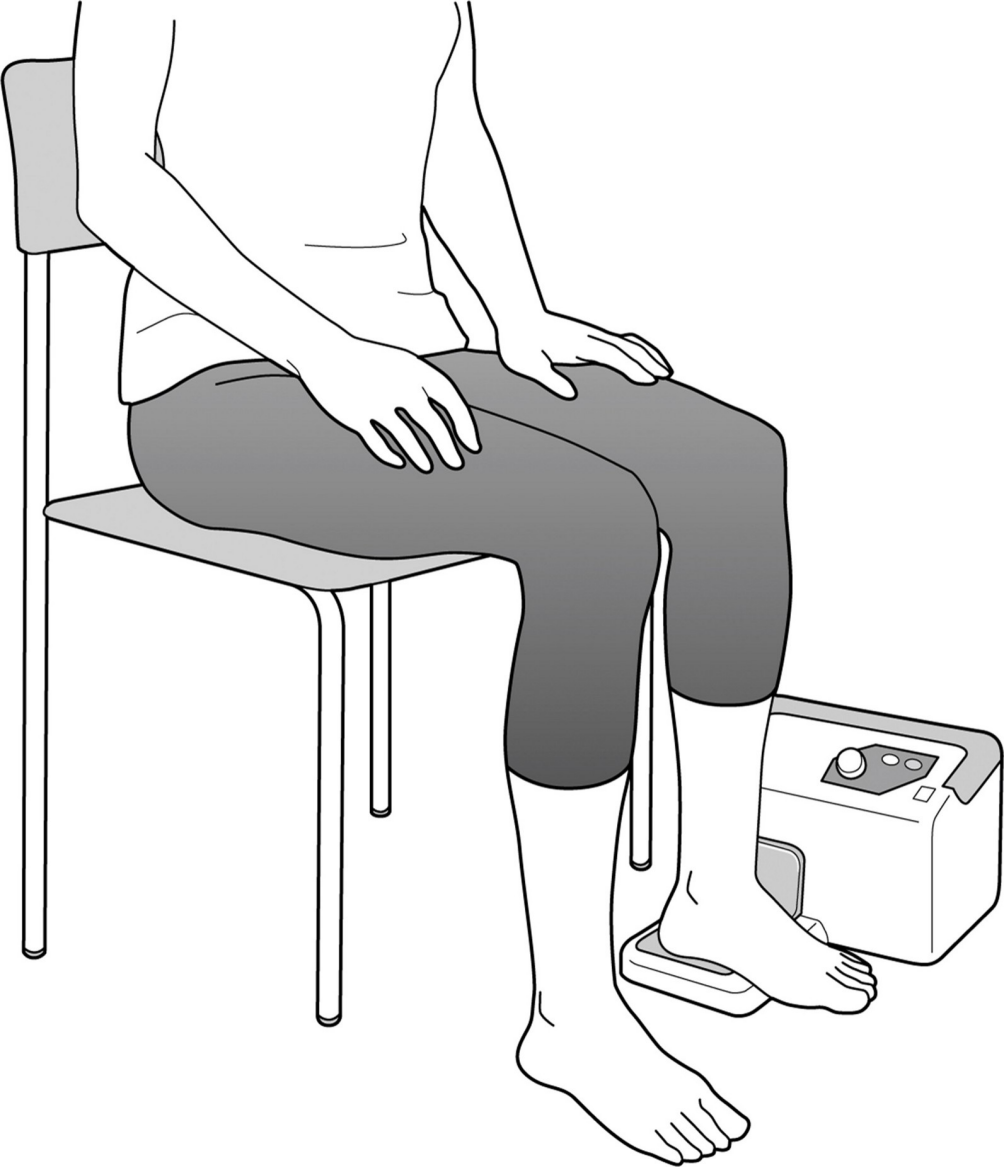
Method of jiggling exercise. KENKO YUSURI® vibrates with the patient’s heel to automate rapid, short, up-and-down movements. The jiggling exercise was performed on a chair in the sitting position to avoid deep flexion of the hip.

Jiggling exercise has been proposed as a new therapeutic exercise for hip OA [8], with reference to the concept of continuous passive motion involving non-strenuous, repetitive joint movements [9]. Jiggling exercise, which involves continuous shaking of the foot and leg in small oscillations (Fig 1), has been reported as the easiest and least invasive treatment for patients with advanced- and terminal-stage hip OA [8]. Some case reports have described the clinical utility of jiggling exercise for hip OA, such as radiographic changes, increases in joint space width (JSW), and remission of symptoms [8, 10–12]. Increased pain in hip OA is strongly associated with reduced JSW values [13–15]; therefore, jiggling exercise may be expected to relieve pain in even inoperable patients or patients with poor postoperative progress. However, all past studies investigating jiggling exercises have been performed in outpatient subjects, most of whom took between 1 to 2 years to show improvement in the JSW [10, 12]. One study on jiggling exercise that investigated short-term clinical outcomes over 3 months indicated a trend of improvement in the scores of the Japanese Orthopaedic Association Hip Disease Evaluation Questionnaire in 48 patients with hip OA; however, the JSW values were not shown [16].

We hypothesized that intensive jiggling exercises for inpatients with hip OA would result in earlier improvement in JSW and pain, which might serve as a disease-modifying non-invasive intervention for hip OA. The purpose of this study was to use the data from our own treatment to evaluate the short-term effectiveness of intensive jiggling exercises in relation to JSW and pain in inpatients with hip OA, to examine the clinical utility of this exercise.

## Materials and methods

This study was a single-center retrospective case series. The protocol followed the tenets of the Declaration of Helsinki and was approved by the ethics committee of the Yanagawa Rehabilitation Hospital, Fukuoka, Japan (authorization number 2019–334). All study participants provided written informed consent. All the patients’ information was anonymized and de-identified prior to analysis.

### Study patients

We retrospectively reviewed the records of all patients with hip OA who performed jiggling exercises at Yanagawa Rehabilitation Hospital (Fukuoka, Japan) in inpatient settings between July 1st, 2017 and January 31st, 2019.

The inclusion criteria were as follows: (1) diagnosis of advanced- and terminal-stage hip OA; (2) some pain related to the hip joint; (3) narrowing radiographic JSW of the hip joint; (4) patient’s desire to avoid surgery; and (5) continuous daily jiggling exercise performed from date of hospital admission to 6 months post-discharge. The exclusion criteria were as follows: (1) bilateral hip OA, (2) extreme decrease in acetabular head index (<50%), (3) history of neurological disease, (4) missing measurements, and (5) loss to follow-up.

We assessed nine hip joints in nine patients. The cohort consisted of three male and six female patients, and their ages at the time of hospital admission ranged from 37 to 71 years (mean ± standard deviation [SD], 57 ± 12 years) (Table 1). Each patient was affected on the right side. The patients’ relevant past medical histories were as follows: (Patient A) articular rheumatism, diabetes mellitus, and ankle OA; (Patient B) diabetes mellitus; (Patient C) osteoporosis, right-sided congenital hip dislocation, and right-sided Chiari pelvic osteotomy; (Patient D) right-sided Chiari pelvic osteotomy; (Patient E) right-sided femoroacetabular impingement; (Patient F) none; (Patient G) right-sided pyogenic arthritis of the hip; (Patient H) right-sided osteonecrosis of the femoral head; (Patient I) left-sided Chiari pelvic osteotomy.

**Table 1:** Details of nine patients with hip osteoarthritis.

Patient number	A	B	C	D	E	F	G	H	I	Mean (SD)
**Age (years)**	38	71	60	64	62	63	37	53	61	56.6 (11.8)
**Sex**	M	F	F	F	F	F	M	M	F	–
**Height (m)**	1.69	1.52	1.52	1.58	1.62	1.50	1.59	1.68	1.61	1.59 (0.07)
**Weight (kg)**	68.0	60.0	61.0	64.5	58.0	46.5	54.0	48.0	46.3	56.3 (8.0)
**Body Mass Index (kg/m**^**2**^**)**	23.8	26.0	26.4	25.8	22.1	20.8	21.4	17.0	17.9	22.4 (3.5)
**Period of hospitalization (days)**	31	27	67	32	62	27	27	98	85	50.7 (27.9)
**Duration of jiggling exercise**										
**In hospitalization (hours/ day)**	2	2	2	2	4	3	5	6	3	3.2 (1.5)
**In post-discharge (hours/ day)**	1	1	1	1	1	1	1	1	1	1.0 (0.0)

SD: standard deviation; M: male, F: female.

### Jiggling exercise

According to Hiromatsu et al. [10], jiggling exercise was performed by the patients themselves using the KENKO YUSURI^®^ (TOPRUN Co., Ltd., Fukuoka, Japan; Fig 1). The patient places their heel on this device, which vibrates at 3.3–5.0 Hz to automate rapid, short, up-and-down movements similar to jiggling one’s knee. At hospital admission, the orthopedic surgeon and physical therapist instructed the patients regarding their sitting posture when performing jiggling exercises on a chair, specifically to avoid deep flexion of the hip joint. Jiggling exercises were recommended to all patients for at least 2 hours per day during hospitalization and for at least 1 hour (preferably 2 hours) per day post-discharge. Each patient was provided with a notebook to keep track of the actual time spent performing the jiggling exercise; this was self-reported by the patients. Additionally, to reduce mechanical stress on the hip joint, the patients were instructed to not perform any weight-bearing while using a wheelchair, and to lose weight through caloric restriction during hospitalization. After discharge, the patients were also instructed to use a cane as much as possible, to not use the stairs, to not gain weight, to not stand up from the floor or sit on the floor, and to not carry heavy objects.

### Measuring methods

This study examined the value of minimum JSW and pain at hospital admission, at discharge, and at the 6-month post-discharge checkup.

**Fig 2:**
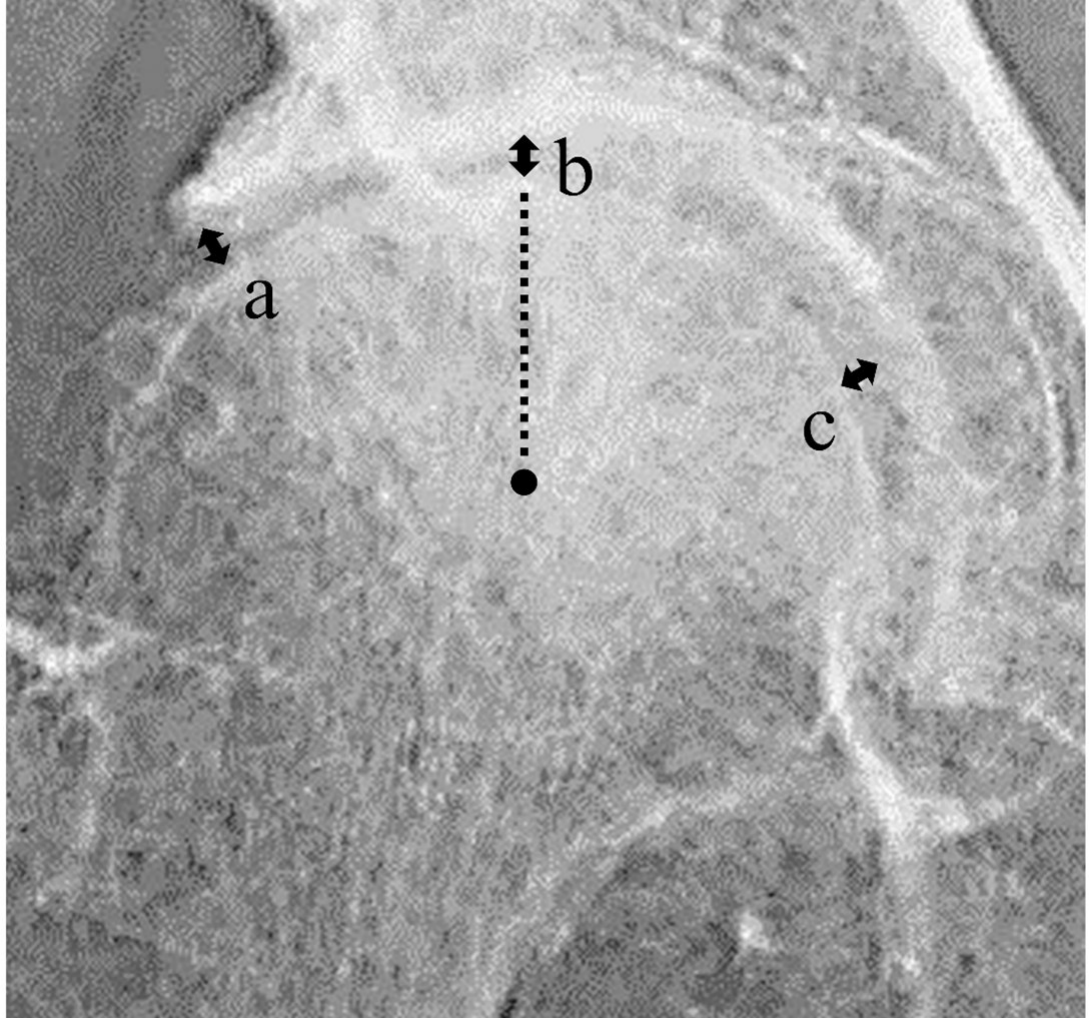
Measurement of the minimum joint space width (JSW). The minimum JSW was measured at three locations: lateral JSW (a), center JSW (b), and medial JSW (c), or at a fourth location if another minimum JSW was observed. Minimum JSW was selected as the smallest measurement.

According to Jacobsen and Sonne-Holm [17], the minimum JSW was measured at three locations: at the lateral margin of the subchondral sclerotic line (lateral JSW), at the apical transection of the weight-bearing surface by a vertical line through the center of the femoral head (center JSW), and at the medial margin of the weight-bearing surface bordering on the fovea (medial JSW); it was measured at a fourth location if the minimum JSW was observed outside the three standard locations (Fig 2). The minimum JSW was selected as the smallest of these three or four measurements. All measurements were performed by two expert physical therapists using a picture archiving and communication system (SOFTMAX Co., Ltd, Tokyo, Japan). The minimum unit of value recorded for JSW was 0.1 mm. The accuracy of the measurement technique was determined by repeating all the measurements a total of three times.

For pain evaluation, the Japanese Orthopaedic Association hip score for pain (JOA hip pain score) was applied as this score is associated with roentgenographic changes in hip OA [18]. This score is recorded on a six-point scale that ranges from 40 (no pain) to 0 (unbearable pain) points and is reportedly a reliable system for patients with hip OA undergoing conservative treatment [19].

## Results

The intervention period (days) and jiggling exercise duration (hours) varied in each case. The period of hospitalization and daily jiggling exercise time ranged from 27 to 98 days and 2 to 6 hours, respectively (Table 1). The daily jiggling exercise duration post-discharge was 1 hour for all nine patients.

**Fig 3:**
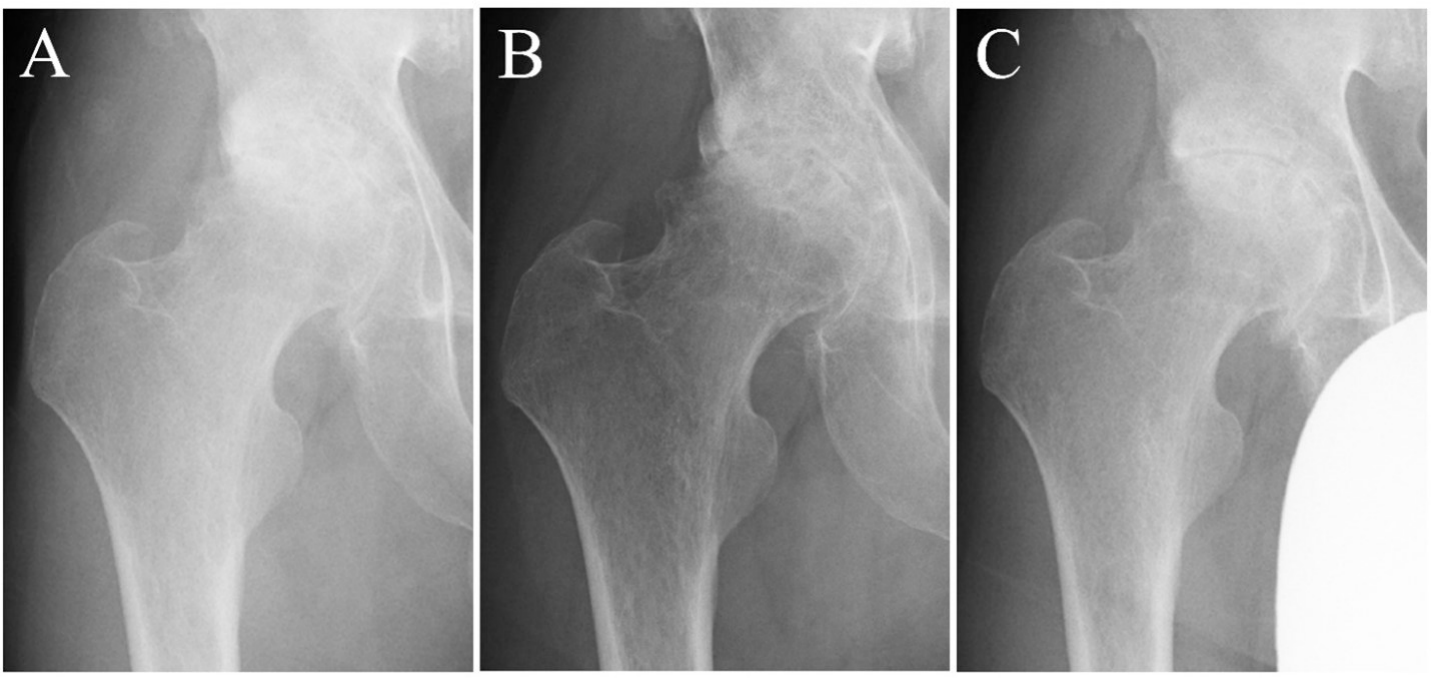
Recording from a single subject (patient A) showing radiographic changes in hip joint. At hospital admission (A), discharge (B), and at the 6-month post-discharge checkup (C).

The records from one patient (patient A) illustrating the radiographic changes in the hip joint at hospital admission, discharge, and at the 6-month post-discharge checkup is presented in Fig 3. The results of the JSW of the hip joint are shown in Table 2. The mean values (range) of minimum JSW at hospital admission, discharge, and 6 months post-discharge were 0.3 ± 0.4 mm (0–1.4 mm), 0.6 ± 0.6 mm (0–1.8 mm), and 0.5 ± 0.4 mm (0–1.1 mm), respectively. The mean value of minimum JSW at discharge was the highest, compared to that at hospital admission and 6 months post-discharge. The minimum JSW had improved in five patients (patients B, C, E, F, and G) at discharge, compared to that at hospital admission (+0.2–0.8 mm), and in four patients (patients A, G, H, and I) at 6 months post-discharge, compared to that at discharge (+0.1–0.9 mm). The minimum JSW continued to increase from hospital admission to 6 months post-discharge in only patient G. In terms of the three locations of the JSW, all nine cases showed increased values at one or more locations. During the period of hospitalization, the maximum increased values were +0.8 mm in minimum JSW (patient C), +1.8 mm in lateral JSW (patient C), +1.3 mm in center JSW (patient A), and +1.5 mm in medial JSW (patient F).

**Table 2:** Results of joint space width of the hip joint in nine patients (mm).

Patient number		A	B	C	D	E	F	G	H	I	Mean (SD)	Range
**Minimum JSW**	At admission	0	1.4	0	0	0.3	0.4	0.4	0.4	0	0.3 (0.4)	0–1.4
	At discharge	0	1.8	0.8	0	0.7	0.7	0.6	0.4	0	0.6 (0.6)	0–1.8
	At 6 months post-discharge	0.2	1.1	0	0	0.7	0.4	0.7	0.5	0.9	0.5 (0.4)	0–1.1
**Lateral JSW**	At admission	0	2.4	0.6	2.4	1.4	4.5	5.3	0.4	0.7	2.0 (1.9)	0–5.3
	At discharge	1.4	3.3	2.4	2.0	0.7	4.6	4.6	0.4	0.9	2.3 (1.6)	0.4–4.6
	At 6 months post-discharge	0.2	3.1	1.5	3.3	2.9	4.6	6.5	0.5	0.9	2.6 (2.1)	0.2–6.5
**Center JSW**	At admission	0	2.5	0.9	0	1.8	1.4	2.8	0.9	0.9	1.2 (1.0)	0–2.8
	At discharge	1.3	3.0	2.1	0	1.8	1.2	1.9	0.9	1.1	1.5 (0.8)	0–3.0
	At 6 months post-discharge	2.0	2.2	2.0	0	1.1	0.9	2.3	0.9	0.9	1.4 (0.8)	0–2.3
**Medial JSW**	At admission	0	2.5	1.0	3.2	1.8	3.0	3.4	4.4	4.1	2.6 (1.4)	0–4.4
	At discharge	0.6	3.4	1.2	3.7	1.9	4.5	3.1	4.2	3.9	2.9 (1.4)	0.6–4.5
	At 6 months post-discharge	0.7	3.6	2.3	3.0	3.7	3.4	4.0	4.6	3.5	3.2 (1.1)	0.7–4.6

JSW: joint space width; SD: standard deviation.

The results of the JOA hip pain scores are presented in Table 3. The mean values (range) at hospital admission, discharge, and 6 months post-discharge were 6.7 ± 7.1 points (0–20 points), 24.4 ± 11.0 points (10–40 points), and 23.3 ± 12.0 points (10–40 points), respectively. The mean score at discharge was the highest compared to that at hospital admission and 6 months post-discharge. Eight patients showed increased scores, whereas only patient I retained the original score after treatment.

**Table 3:** Results of the Japanese Orthopaedic Association hip score for pain in nine patients (points).

Patient number	A	B	C	D	E	F	G	H	I	Mean (SD)	Range
**At admission**	0	0	20	0	0	10	10	10	10	6.7 (7.1)	0–20
**At discharge**	20	35	35	40	10	30	20	20	10	24.4 (11.0)	10–40
**At 6 months post-discharge**	30	35	20	40	10	35	20	10	10	23.3 (12.0)	10–40

Full marks = 40 points. Forty points (None): No pain and/or no complaints related to the hip joint. Thirty-five points (Ignores): no pain with inconsistent symptoms, including feelings of weariness or dullness. Thirty points (Slight): No spontaneous pain, with some pain when walking (including slight pain when starting to walk or after walking for long distances). Twenty points (moderate): no spontaneous pain, with some pain when walking that disappears quickly after a short rest. Ten points (severe): spontaneous pain that is severe when attempting to walk and decreases after rest. Zero points (unbearable): continuous pain during rest and/or at night. SD: standard deviation.

## Discussion

This is the first study to examine short-term minimum JSW changes in inpatients with advanced- and terminal-stage hip OA after intensive jiggling exercise. Our results suggested that intensive jiggling exercises may facilitate early improvement in pain and radiographic JSW values.

A previous study on jiggling exercise for outpatients with hip OA has reported that the JSW increased in 65 of 92 patients with post-Chiari pelvic osteotomy (70.7%) [10]. The authors also noted that an increase in JSW by about 2.0 mm took around 2 years. In another study, Teramoto et al. [12] reported that two patients with advanced- and terminal-stage hip OA who performed jiggling exercise showed remarkable clinical improvement and some improvement in joint congruity that was observed on hip radiographs. The authors also noted that the radiographic improvement in joint congruity took longer than 1 year. In contrast, our study showed that with jiggling exercise performed through 27–98 days of hospitalization, there was not only an increase in the minimum JSW in five inpatients (55.6%), but an increase in the JSW in all nine inpatients in one or more locations. The increased JSW values obtained in this study corresponded to those reported by Hiromatsu et al. [7] (1.8 mm vs. 2.0 mm).

The increased value of the JSW may have been caused by a decrease in muscle tension around the hip joint. A previous study has reported that 10 minutes of jiggling exercise using a KENKO YUSURI^®^ significantly reduces muscular stiffness of the gluteus maximus and biceps femoris muscles in patients with hip OA [11]. Thus, similar changes in muscle tension around the hip joint may have occurred in the patients of our study. In addition, it was reported that higher daily cumulative hip moment was a predictor of radiographic progression of hip OA [20]. It is presumed that the reduction of mechanical stress and muscle activity around the hip joint by not bearing weight also influenced JSW changes. However, our results showed that locations with increased JSW differed from case to case. Chiari pelvic osteotomy has been reported to reduce the gluteus medius abductor torque [21]. Due to a medical history of right-sided Chiari pelvic osteotomy, patients C, D, and I showed an increase in lateral JSW. Past medical histories may have influenced JSW improvement.

The JOA hip pain score increased in eight patients (88.9%) at discharge (+10–40 points). Regarding the relationship between the minimum JSW and the hip pain, previous studies have reported that hip OA pain is associated with a minimum JSW of <2.0 mm [14] or <1.5 mm [22]. In this study, the minimum JSW in all nine patients at discharge was <1.8 mm; among them, three patients had a JSW of 0 mm. Patient D, in particular, reported an improvement in the JOA hip pain score up to full 40 points, despite the fact that the minimum JSW remained at 0 mm from hospital admission to 6 months post-discharge. Therefore, a consistent relationship between the two parameters was not observed in this study. With the improvement of not only the minimum JSW, but also the JSW in either of the three locations, the distribution of mechanical stress on the weight-bearing surface may have led to a reduction in pain.

While all nine patients continued the jiggling exercise for up to 6 months post-discharge, the mean value of the minimum JSW at discharge was the highest. The daily jiggling exercise duration for nine patients ranged from 2 to 6 hours daily during hospitalization, and 1 hour daily post-discharge. Thus, it may be desirable to conduct jiggling exercises for at least 2 hours daily to increase the JSW. Each patient was expected to return to independent living following discharge; therefore, although they were instructed to perform jiggling exercises for preferably 2 hours daily, they were realistically expected to do so for at least 1 hour. The result was 1 hour of jiggling exercise performed daily by all patients following discharge. The fact that the patients accepted hospitalization for the jiggling exercise indicates that they were highly motivated to perform the exercise. This high level of motivation may have been influenced by the patient’s desire to improve the severity of their pain, which may have been difficult to maintain following discharge when the pain had improved or reduced. In addition, the combination of jiggling exercise and non-weight-bearing during hospitalization may have been effective as well. However, patient H, who had the longest hospital stay and daily duration of jiggling exercise, showed no change in minimum JSW at discharge. It is necessary to further examine the factors that lead to the improvement in the minimum JSW.

### Limitations

This study has some limitations. First, although there were several potential patients at the beginning of this study, the number of patients decreased due to the stringent inclusion/exclusion criteria. It was, however, crucial to have a homogeneous group of patients with hip OA to precisely evaluate the short-term effectiveness of intensive jiggling exercise. Second, as the intervention period (days) and daily jiggling exercise duration (hours) varied in each case, a statistical analysis could not be performed. Third, detailed information on the combined effects of jiggling exercise and non-weight-bearing remains unclear. A randomized controlled trial is required to confirm the same. Fourth, the relationship between increased JSW and walking ability could not be examined. Future investigations on changes in walking and daily routine activities are also required.

## Conclusions

Our results suggested that intensive jiggling exercises for inpatients with advanced- and terminal-stage hip OA facilitated early improvement in radiographic JSW and pain. Daily jiggling exercise for a sufficient duration or a combination of this therapy and non-weight-bearing might be a feasible conservative treatment for hip OA.
